# Feasibility of large language models for assessing and coaching surgeons’ non-technical skills

**DOI:** 10.1038/s44401-025-00027-2

**Published:** 2025-07-15

**Authors:** Marian Obuseh, Sneha Singh, Nicholas E. Anton, Robin Gardiner, Dimitrios Stefanidis, Denny Yu

**Affiliations:** 1 Edwardson School of Industrial Engineering, Purdue University, West Lafayette, IN USA; 2 Koita Centre for Digital Health, Indian Institute of Technology, Bombay, India; 3 School of Medicine, Indiana University, Indianapolis, IN USA

**Keywords:** Health care, Machine learning

## Abstract

This study demonstrates Large Language models (LLMs) to assess and coach surgeons on their non-technical skills, traditionally evaluated through subjective and resource-intensive methods. Llama 3.1 and Mistral effectively analyzed robotic-assisted surgery transcripts, identified exemplar and non-exemplar behaviors, and autonomously generated structured coaching feedback to guide surgeons’ improvement. Our findings highlight the potential of LLMs as scalable, data-driven tools for enhancing surgical education and supporting consistent coaching practices.

Large language models (LLMs) have demonstrated strong performance in general natural language processing (NLP) tasks across various domains^1^. In healthcare, studies have shown promising results particularly in medical diagnostic support and clinical decision-making^2,3^. For example, GPT-4 achieved a diagnostic accuracy of 61.1% in its top six differential diagnoses for previously unpublished challenging clinical cases, surpassing the 49.1% accuracy of physicians^2^. Similarly, a Llama-based model achieved 52% accuracy in assigning diagnosis-related groups for hospitalized patients using clinical notes^3^. These advancements highlight the potential of LLMs in specialized healthcare applications, including skill assessment and training of healthcare professionals. While substantial work has focused on assessing and training surgeons’ technical skills^4,5^, there is less emphasis on non-technical skills (NTS)^6^. However, these NTS are critical cognitive, social, and interpersonal abilities that are directly linked to adverse events and patient safety outcomes^7^.

Assessing NTS presents unique challenges due to their primary expression through verbal communication^8^, which can be dynamic, nuanced, context-dependent, and susceptible to interpretation bias. This variability introduces subjectivity into assessments, as observers may interpret the same behaviors differently. Traditional observational assessments are prone to these inconsistencies that can compromise subsequent training efforts. They are also resource-intensive, requiring significant time and effort not only to train the raters but also for the raters to conduct the assessments for long surgeries^9,10^. Dyadic NTS coaching approaches, while beneficial, demand specialized non-surgeon coaching staff who can focus less on technical skills and are also very time-intensive^11,12^. Given these challenges, there is a clear need for more objective, efficient, and scalable methods to assess NTS, ensuring consistent, high-quality training that enhances patient outcomes and strengthens team dynamics.

NLP offers a promising solution to these challenges since NTS are mostly demonstrated through verbal communication. In this study, we explore the feasibility of LLMs for surgeons’ NTS assessment and coaching. Specifically, we hypothesize that LLMs can differentiate between exemplar and non-exemplar NTS behaviors and provide actionable performance coaching feedback to surgeons, supporting scalable and effective surgical education.

## Model performance

Table 1 presents the performance of LLMs and benchmarking machine learning (ML) models in categorizing NTS behaviors. Overall, LLMs demonstrated higher performance than classical ML models. Llama 3.1 achieved a macro-averaged F1 score of 0.62 and a prediction accuracy of 74%, while Mistral showed comparable performance with a macro-averaged F1 score of 0.61 and a prediction accuracy of 75%. In comparison, the classical ML models including logistic regression (LR) and support vector machines (SVM), demonstrated lower overall performance. Although their prediction accuracy (LR = 74%, SVM = 71%) was comparable to LLMs, their macro-averaged F1 scores were significantly lower (LR = 0.52, SVM = 0.51), highlighting their limitations in balancing precision and recall across NTS behaviors.

**Table 1:** Overall and class performance on test data for each model category

Overall performance on test data for each model category					
Model category	Model name	Performance metrics			
		Accuracy	Precision	Recall	F1 Score
Classical ML	LR	0.74	0.55	0.71	0.52
	SVM	0.71	0.55	0.73	0.51
LLM	Llama 3.1	0.74	0.61	0.66	0.62
	Mistral	0.75	0.60	0.63	0.61
**Class performance on test data for each model category**					
Model category	Model name	NTS performance rating	Performance metrics		
			Precision	Recall	F1 Score
Classical ML	LR	Exemplar	0.98	0.75	0.85
	SVM		0.98	0.71	0.83
LLM	Llama 3.1		0.90	0.78	0.84
	Mistral		0.88	0.81	0.84
Classical ML	LR	Non-exemplar	0.11	0.67	0.19
	SVM		0.11	0.75	0.19
LLM	Llama 3.1		0.33	0.54	0.41
	Mistral		0.32	0.45	0.37

F1 scores (harmonic mean of precision and recall) regarded as most important metric.

*LLM* large language model, *ML* machine learning, *LR* linear regression, *SVM* support vector machine, *NTS* non-technical skills.

For exemplar NTS behaviors, both LLMs and classical ML models performed excellently, with macro-averaged F1 scores of 0.84 and 0.85 respectively. This indicates that both model types were effective in identifying well-defined, positive behaviors exhibited by surgeons. For non-exemplar behaviors, which are more challenging to identify, Llama 3.1 achieved a macro-averaged F1 score of 0.41, outperforming LR and SVM, both of which had F1 scores below 0.20. Mistral also performed better than the classical models with an F1 score of 0.37. These results show that while classical ML models maintained comparable performance for exemplar behaviors, LLMs provided improved accuracy and F1 scores for non-exemplar behavior identification.

**Fig. 1:**
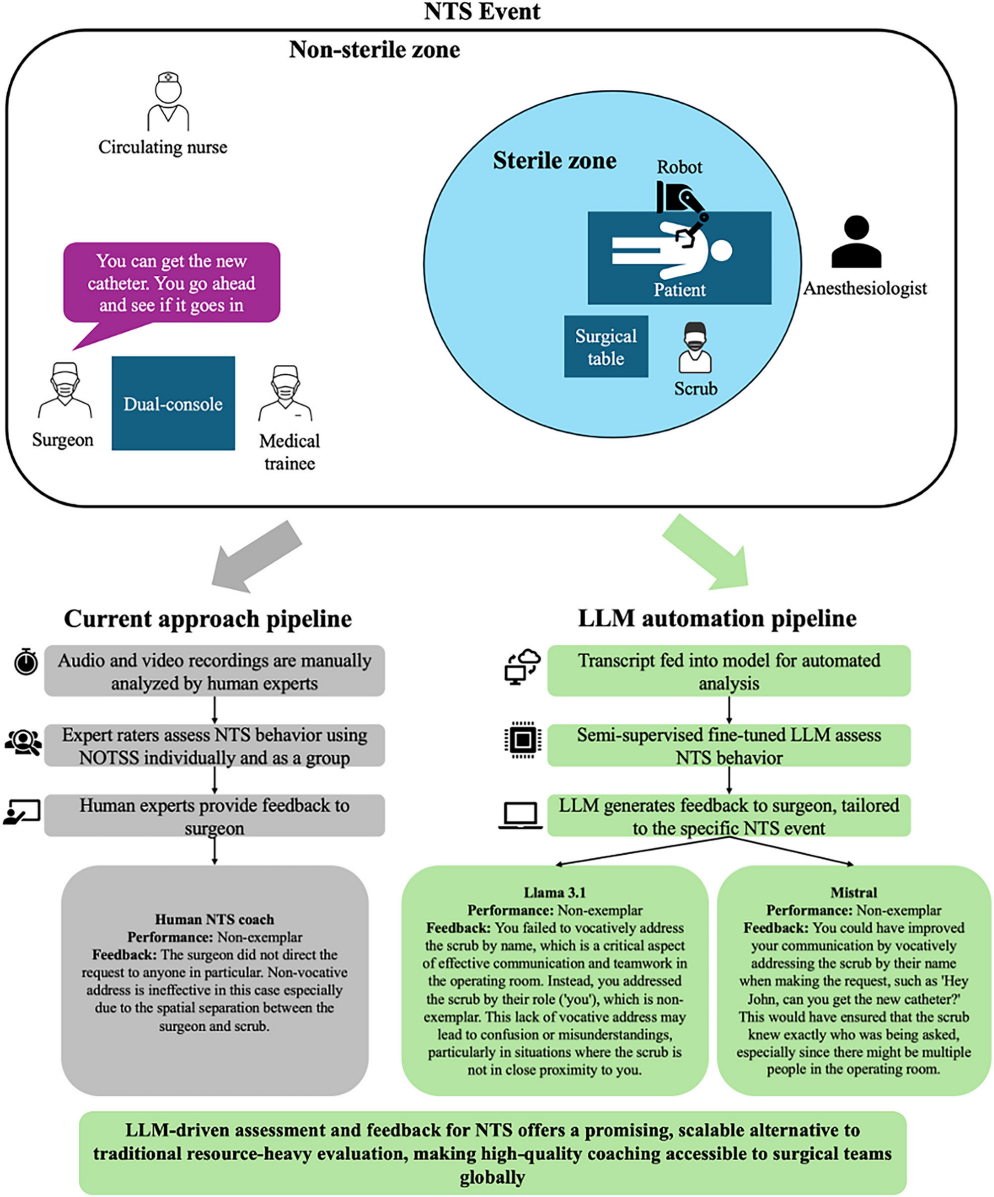
Comparative workflow for current gold standard versus LLM-based approach for NTS assessment and coaching for a surgical event. Classical ML are excluded, as they do not support natural language generation.

Figure 1 compares the traditional manual NTS assessment pipeline with the proposed LLM-based automation approach using an example of a missed communication opportunity in the operating room due to the lack of a vocative address. In the manual approach^12^, multiple human expert raters, including a coach, independently assess the surgeon’s NTS behaviors using the Non-Technical Skills for Surgeons (NOTSS) tool. After completing the individual assessments, they engage in group discussions to reach a consensus on the assessment. The coach then provides dyadic coaching feedback to the surgeon, specifically targeting improvements to address the identified communication breakdown. This multi-step process is resource-intensive, requiring substantial time for individual assessments, consensus-building, and personalized coaching.

In contrast, the LLM automation pipeline directly processes surgical transcripts through fine-tuned models to assess NTS behaviors and generate coaching-style feedback. Both Llama 3.1 and Mistral identified the same non-exemplar behavior in the example scenario. Llama 3.1 highlighted potential confusion due to the lack of a vocative address, while Mistral offered specific suggestions for clearer communication. Additional examples of exemplar and non-exemplar classifications can be found in Supplementary Table S1. These results demonstrate that LLMs can automate NTS coaching, providing consistent, context-specific feedback that aligns with human coaches while offering a more efficient and scalable alternative to manual methods.

## Implications

This study demonstrated the feasibility of LLMs for NTS assessment and coaching in surgeries, addressing key limitations of traditional methods. Our results showed that LLMs outperformed ML models despite being trained on significantly smaller datasets. Differences in data handling strategies should be considered when interpreting classification performance comparisons. Although balanced training data were used in both cases to mitigate class imbalance effects, the underlying training mechanisms differed.

LLM performance was likely constrained by limited context windows, which restricted the amount of contextual information that could be processed during training^13^. In particular, the shorter context window of Mistral limited the size of the training set. While this enabled consistent evaluation across LLMs and a focus on developing highly targeted prompts, it may reduce generalizability. This limitation should be revisited as LLMs advance. Additionally, the underrepresentation of non-exemplar behaviors in the dataset likely contributed to lower performance in this category. In this study, fewer than 17% of observed behaviors were non-exemplar, aligning with prior research indicating that while these behaviors are less frequent, they are not uncommon^14^. Ongoing research is focused on enhancing model adaptability through optimized fine-tuning strategies and dataset expansion, particularly by increasing non-exemplar cases to improve class balance.

Despite not being explicitly trained on human coaching feedback, LLMs demonstrated the ability to generate performance-based feedback for surgeons, offering a scalable alternative to traditional dyadic human coaching, which is often limited by human availability and variability. This scalability could democratize access to high-quality NTS training, benefiting a wide range of surgical teams, including those in resource-limited settings. The ability to better identify non-exemplar behaviors, where coaching is most needed, combined with the generation of structured, context-specific feedback, makes LLMs preferable in this setting.

This study focused on transcript-based, offline analysis that lays critical groundwork for future efforts by (1) establishing a reproducible pipeline for building large-scale datasets from real intraoperative conversations, (2) demonstrating the advantages of LLMs over traditional ML models in capturing the nuance of verbal behaviors during RAS, (3) refining prompt engineering strategies to handle domain-specific language and achieve high performance on NTS performance classification and coaching feedback tasks, and (4) producing a trained LLM-based model that can be further expanded, refined, validated, and adapted. Early-stage large-scale human evaluation of LLM-generated feedback will be a formal part of the system design, with expert review helping to shape model refinement. Initial feedback efforts will focus on acceptability, perceived usefulness, trust, and integration into training workflows. A full usability study will be necessary before deployment to systematically evaluate acceptability, trust, perceived usefulness, and workflow integration in clinical environments. Practical implementation will require addressing barriers such as generalizability across specialties and institutions, alignment with clinical education standards, and fidelity to the dynamic context of live procedures. It will be essential to address concerns around accountability, reliability, privacy, and the secure handling of clinical data to prevent misuse of sensitive information. Additionally, we recognize that LLMs operate as black-box systems, which presents challenges for interpretability and adoption. A human-in-the-loop approach will therefore remain essential throughout the design, development, deployment, and post-deployment monitoring phases. While automation reduces the overall burden compared to traditional dyadic coaching, expert oversight by surgical educators or system stakeholders will remain essential to ensure accountability. Together, these efforts aim to enable scalable, targeted, and high-fidelity NTS coaching while establishing a clearer pathway toward integrating LLM-based feedback mechanisms into surgical education programs.

## Methods

### Participants

This study was approved by Indiana University’s Institutional Review Board (protocol 1702481748; approved: March 2023). No race, ethnicity, or patient-identifiable data was collected. Between 2023 and 2024, nine surgeons (4 women [44%]) from six surgical subspecialties across three hospitals in Indiana University Health system, all performing at least one robotic-assisted surgery (RAS) per month, were recruited. They provided informed consent, and twenty RAS were observed and recorded in the operating room, with each surgeon contributing 1–3 cases.

### Data

Human expert raters watched the RAS recordings and used the validated NOTSS^15^ tool to identify operating room interactions that challenged the surgeons’ NTS. Surgeons’ performance was classified as exemplar (rating = 4) or non-exemplar (rating < 4). This classification approach aligns with prior research distinguishing behaviors meeting the highest standard from those requiring improvement^9^. The raters identified 709 NTS events, with 590 categorized as exemplar behaviors and 119 as non-exemplar. Transcripts of these interactions constitutes the dataset.

### Large language models

This study used publicly released state-of-the-art models, including Meta’s Llama-3.1-8B-Instruct^16^ (open-weight, under a custom non-commercial license) and Mistral AI’s Mistral-7B-Instruct-v0.3^17^ (open-weight and open-source under Apache 2.0), both accessed via Hugging Face. All experiments were conducted using Google Colab Pro+, which provided cloud-based access to a single A100 GPU. The only cost incurred was the Colab Pro+ subscription, which offered sufficient compute capacity for prompt engineering, model inference, and performance evaluation. These models were chosen for their strong performance, computational efficiency, and accessibility, which enable execution within controlled research environments without reliance on proprietary API services. This supports reproducibility, transparency, and privacy, which are key considerations for clinical research.

While Llama 3.1 supports a context window of up to 128 K tokens^16^, our experiments were constrained by Mistral’s shorter context window of approximately 32 K tokens^17^. This limitation necessitated a focus on developing highly targeted prompts rather than increasing the size of the training dataset. A stratified sampling strategy was used to construct the training dataset. One exemplar and one non-exemplar data point were randomly sampled from each of the four NOTSS categories, resulting in eight data points (~1% of data), covering all combinations of NTS performance level and construct. The same training data (*n* = 4 exemplar class and *n* = 8 non-exemplar class) was used for both LLMs.

Prompt design targeted both classification of NTS performance and generation of coaching feedback. For both models, the generation parameters were as follows: max_new_tokens = 512 (prevents overly long coaching feedback), temperature = 0.005 (makes the output consistent across runs), top *p* = 0.001 (restricts choices to only the most likely words), top *k* = 20 (adds a cutoff to avoid unusual or irrelevant words), and do sample = True (to retain some natural language variability). Importantly, the models were not trained on any human coaching feedback. Instead, they were prompted to generate feedback solely from the transcripts of surgeon interactions in the operating room, ensuring that feedback generation was independent of expert coaching examples.

To evaluate model performance with minimal instruction, an initial prompt was executed and outputs were manually reviewed to identify failure patterns. Prompts were subsequently refined iteratively using evidence-based strategies, including role prompting (e.g., “You are an experienced non-technical skills coach”), clear section delimitation, and zero-shot chain-of-thought prompting (e.g., “Think step-by-step”)^18,19^. Refinement continued until both models achieved perfect (100%) classification and feedback generation performance on the training data. The final prompts can be found in the Supplementary Information. Model performance was evaluated on the full test dataset (*n* = 701; 115 non-exemplar) using classification metrics including accuracy, precision, recall, and macro-averaged F1 scores.

### Classical machine learning models

Classical ML models (Linear Regression and Support Vector Machine) were used as benchmarks for evaluating LLM performance. To address class imbalance, the majority class (exemplar) was under-sampled to 80% of the minority class^20^, creating a balanced training dataset (*n* = 190; 95 non-exemplar). The models were trained on Term Frequency-Inverse Document Frequency (TF-IDF) vectorized unigrams of the balanced training data. Model performance was then evaluated on a held-out test dataset (*n* = 519; 24 non-exemplar) using the metrics as the LLMs.

## Supplementary information

Supplementary Information

## Data Availability

Due to Indiana University ethics policy and to protect participants privacy, the data used in this study cannot be publicly shared. However, sample de-identified datapoints are provided in the Supplementary Information.

## Code availability

Due to Indiana University ethics policy and to protect participants privacy, the full codes used in this study cannot be publicly shared. However, partial model prompts are provided in the Supplementary Information.

## References

[1] Raiaan, M. A. K. et al. A review on large language models: architectures, applications, taxonomies, open issues and challenges. *IEEE Access* **12**, 26839–26874 (2024).

[2] Rutledge, G. W. Diagnostic accuracy of GPT-4 on common clinical scenarios and challenging cases. *Learn Health Syst* **8**, e10438 (2024). 39036534 10.1002/lrh2.10438 PMC11257049

[3] Wang, H., Gao, C., Dantona, C., Hull, B. & Sun, J. DRG-LLaMA: tuning LLaMA model to predict diagnosis-related group for hospitalized patients. *npj Digital Med.* **7**, 1–9 (2024). 10.1038/s41746-023-00989-3 PMC10803802 38253711

[4] Lam, K. et al. Machine learning for technical skill assessment in surgery: a systematic review. *npj Digital Med.* **5**, 1–16 (2022). 10.1038/s41746-022-00566-0 PMC8894462 35241760

[5] Bjerrum, F., Thomsen, A. S. S., Nayahangan, L. J. & Konge, L. Surgical simulation: current practices and future perspectives for technical skills training. *Med. Teach.* **40**, 668–675 (2018). 29911477 10.1080/0142159X.2018.1472754

[6] Mahendran, V., Turpin, L., Boal, M. & Francis, N. K. Assessment and application of non-technical skills in robotic-assisted surgery: a systematic review. *Surg. Endosc* **38**, 1758–1774 (2024). 38467862 10.1007/s00464-024-10713-1 PMC10978706

[7] Flin, R. & O’Connor, P. *Safety at the sharp end. safety at the sharp end*, 10.1201/9781315607467 (CRC Press, 2017).

[8] Yule, S., Flin, R., Paterson-Brown, S., Maran, N. & Rowley, D. Development of a rating system for surgeons’ non-technical skills. *Med. Educ.* **40**, 1098–1104 (2006). 17054619 10.1111/j.1365-2929.2006.02610.x

[9] Cha, J. S. et al. Objective nontechnical skills measurement using sensor-based behavior metrics in surgical teams. *Hum. Factors*, 10.1177/00187208221101292 (2022). 10.1177/00187208221101292 35610959

[10] Cha, J. S. & Yu, D. Objective measures of surgeon nontechnical skills in surgery: a scoping review. *Hum. Factors* **00**, 18720821995319 (2021). 10.1177/0018720821995319 33682476

[11] Granchi, N. et al. Coaching to enhance qualified surgeons’ non-technical skills: a systematic review. *Brit. J. Surg.* **108**, 1154–1161 (2021). 34476480 10.1093/bjs/znab283

[12] Obuseh, M. et al. Development and application of a non- technical skills coaching intervention framework for surgeons: a pilot quality improvement initiative. *PLoS One* **19**, 1–14 (2024). 10.1371/journal.pone.0312125 PMC11548760 39514593

[13] Press, O., Smith, N. A. & Lewis, M. Train short, test long: attention with linear biases enables input length extrapolation. In: *ICLR 2022 - 10th International Conference on Learning Representations* (ICLR, 2021).

[14] Yule, S., Flin, R., Paterson-Brown, S. & Maran, N. Non-technical skills for surgeons in the operating room: a review of the literature. *Surgery* **139**, 140–149 (2006). 16455321 10.1016/j.surg.2005.06.017

[15] Yule, S. et al. Surgeons’ non-technical skills in the operating room: reliability testing of the NOTSS behavior rating system. *World J. Surg.* **32**, 548–556 (2008). 18259809 10.1007/s00268-007-9320-z

[16] Dubey, A. et al. The Llama 3 herd of models. arXiv preprint arXiv:2407.21783 (2024).

[17] Jiang, A. Q. et al. Mistral 7B. arXiv preprint arXiv:2310.06825 (2023).

[18] Liu, P. et al. Pre-train, prompt, and predict: a systematic survey of prompting methods in natural language processing. *ACM Comput. Surv.* **55**, 1–35 (2023).

[19] Wei, J. et al. Chain-of-thought prompting elicits reasoning in large language models. *Adv. Neural Inf. Process Syst*. **35**, 24824–24837 (2022).

[20] Van Hulse, J., Khoshgoftaar, T. M. & Napolitano, A. Experimental perspectives on learning from imbalanced data. *ACM Int. Conf. Proc. Ser.* **227**, 935–942 (2007).

