# Supplementary material for: Feasibility of large language models for assessing and coaching surgeons’ non-technical skills
Source: Npj Health Syst. 2025 Jul 15;2:25. doi: 10.1038/s44401-025-00027-2 (PMC12263418; doi:10.1038/s44401-025-00027-2)
Supplement: Supplementary file 1 — Supplementary Information [file 44401_2025_27_MOESM1_ESM.docx]

**Supplementary Information**

This supplementary material has been provided by the authors to give readers additional information about the work.

**Prompt**

"""

## TASK OVERVIEW ##

You're an experienced non-technical skills coach in the medical field tasked with assessing non technical skills of surgeons performing robotic-assisted surgeries in operating rooms.

You will be given transcripts from several scenarios recorded during these surgeries along with their corresponding categories.

Your task is to classify the surgeons non technical skills performance for each scenario as exemplar or non-exemplar. You should think step-by-step, having a sound thought process prior to your non-technical skills performance categorization.

Provide a single explanation for the performance rating you chose for that scenario. These explanations will serve as feedback to the surgeon in future coaching sessions. Make the feedback presented in coaching style. Ensure that your explanation highlights specific behaviors that led to your classification, especially non-exemplar behaviors, and offers suggestions for improvement.

Below is the list of categories accompanying the transcripts and what each mean according to the guidance provided by the non-technical skills for surgeon (NOTSS) framework:

<NOTSS FRAMEWORK>

## IDENTIFYING EXEMPLAR AND NON-EXEMPLAR BEHAVIORS ##

## EXAMPLE SCENARIOS AND HELPFUL CONTEXT ##

Here are some sample scenarios.

<GUIDE MODEL WITHOUT PROVIDING EXPLICIT GROUND THRUTH>

<TRAINING DATA POINT 1>

<REPEAT FOR SEVEN OTHER TRAINING DATA POINTS>

## TASK INSTRUCTIONS ##

Let's think step-by-step for each scenario. Given the scenarios, fully understand what's going on in the scenario and the surgeon's thought process, actions, and behaviors. Your task is to:

1. Analyze the Scenario: Carefully read the transcript to understand the context and actions.

2. Identify Behaviors: Note specific behaviors exhibited by the surgeon. Refer to IDENTIFYING EXEMPLAR AND NON-EXEMPLAR BEHAVIORS section.

3. Classify Performance: Decide whether the performance is exemplar or non-exemplar based on the analysis.

4. Provide Explanation: Offer a detailed, coaching-style explanation supporting your classification.

You must output a JSON object which is an array containing an NTS performance rating and an explanation of why the performance rating was chosen for that instance. Do not include any additional text outside of the JSON object.

Here's two examples of what the output format should look like:

[{"performance": "exemplar", "explanation": "You did ..."}]

[{"performance": "non-exemplar", "explanation": "You did not ..."}]

"""

**Models**

1. Llama 3.1-8B-Instruct (Meta AI): <https://huggingface.co/meta-llama/Llama-3.1-8B-Instruct>
2. Mistral-7B-Instruct-v0.3 (Mistral AI): <https://huggingface.co/mistralai/Mistral-7B-Instruct-v0.3>

**Table S1. Examples of exemplar and non-exemplar NTS behaviors**

| Transcripts | Class | Llama 3.1 coaching feedback |
| --- | --- | --- |
| Surgeon: I always make all the marks where I think it's going to be [uses hand to guide where to make marks].  Trainee: But do you agree it's here or is it too lateral? [points]  Surgeon: Let's see [lightly kneads patient stomach]. Yeah I agree. Her eleven is probably like here [points and marks spot] She's like a little harder to feel. So probably like approximately there [makes marking]. So yeah I would say here to start [makes marking], here would be our second [makes marking], here's our third [makes marking], and here's our fourth, approximately [makes marking]! And then I would just make these incisions. This way, you're not just poking her in some random places.  Trainee: Okay.  Scrub: That's fair. All about being deliberate.  Surgeon: Yeah | Exemplar | You effectively communicated with the trainee by asking for their input and validating their concerns, which demonstrates a collaborative approach to decision-making. Additionally, you provided clear and deliberate guidance on where to make the marks, which shows a high level of situation awareness and attention to detail. Your explanation of the reasoning behind your decisions also helps to establish a shared understanding with the team, which is essential for effective communication and teamwork. |
| Surgeon: Okay good. [looks to trainee working by patient's <info de-identified>] How do you feel about things below?  Trainee: Doing good!  Surgeon: Wonderful! [looks around] I feel excellent if you feel good. |  | You effectively checked in with the trainee to gauge their comfort level and confidence in the procedure, which is a great leadership behavior. By asking how they feel, you're showing that you value their input and are willing to adjust your approach based on their feedback. This kind of open communication and teamwork is essential for a successful operation. |
| Nurse: Alright, this is timeout. [surgeon suspends all activities] This is [patient name and date of birth]. She's consented for a robotic <info de-identified> [continues with rest of the timeout]. Uh, fire risk? 2.  Surgeon: Alcohol prep and regenerator?  [nurse gives a thumbs up of affirmation]  Surgeon: Alright, sounds good! | Non-exemplar | You did not conduct a thorough team introduction during the timeout. While you did suspend all activities, you did not ask the team to introduce themselves or their roles, which is an important aspect of ensuring a shared understanding and promoting a safe operating environment. |
| Trainee: [administering local anesthesia] I'm going to go further laterally.  Surgeon: If you want. |  | You did not clearly communicate your plan or expectations to the trainee, which could lead to confusion or miscommunication. In decision-making, it's essential to clearly articulate your thoughts and intentions to the team. In this case, you could have said something like, 'I'd like you to go further laterally with the local anesthesia' to ensure the trainee understands your plan. |
